# Rheumatoid Arthritis Was Negatively Associated with Alzheimer’s Disease: A Population-Based Case-Control Study

**DOI:** 10.1371/journal.pone.0168106

**Published:** 2016-12-20

**Authors:** Li-Ting Kao, Jiunn-Horng Kang, Herng-Ching Lin, Chung-Chien Huang, Hsin-Chien Lee, Shiu-Dong Chung

**Affiliations:** 1 Graduate Institute of Life Science, National Defense Medical Center, Taipei, Taiwan; 2 Research Center of Sleep Medicine, College of Medicine, Taipei Medical University, Taipei, Taiwan; 3 Department of Physical Medicine and Rehabilitation, Taipei Medical University Hospital, Taipei, Taiwan; 4 Department of Health Care Administration, Taipei Medical University, Taipei, Taiwan; 5 Department of Psychiatry, Taipei Medical University-Shuang-Ho Hospital, Taipei, Taiwan; 6 Department of Psychiatry, School of Medicine, College of Medicine, Taipei Medical University; 7 Department of Surgery, Far Eastern Memorial Hospital, Ban Ciao, Taipei, Taiwan; 8 Graduate Program in Biomedical Informatics, College of Informatics, Yuan-Ze University, Chung-Li, Taiwan; Duke University, UNITED STATES

## Abstract

Some of the prior literature investigated the potential association between rheumatoid arthritis (RA) and Alzheimer's disease (AD) because these two diseases may share similar inflammatory mechanisms. Nevertheless, to date, findings of the previous literature are still controversial, and some methodological limitations were observed in those studies. The aim of this case-control study was to investigate the relationship between prior RA and AD using a large population-based dataset. This study used the Taiwan Longitudinal Health Insurance Database 2005. We included 2271 patients with AD who had received prescriptions for acetylcholinesterase inhibitors (AChEIs) as cases and 6813 patients without AD as controls in this study. In addition, we performed a conditional logistic regression to examine the odds ratio (OR) and 95% confidence interval (CI) for prior RA between cases and controls. The study found that 330 (3.63%) of the total sampled patients had an RA diagnosis before the index date. Additionally, prior RA was found in 60 (2.64%) cases and in 270 (3.96%) controls. The conditional logistic regression analysis showed that the crude OR of prior RA for cases was 0.66 (95% confidence interval (CI): 0.49~0.87) compared to controls. After adjusting for patients’ geographic location, urbanization level, and comorbidities, the adjusted OR of prior RA for patients with AD was 0.73 (95% CI: 0.55~0.98) compared to those without AD. We concluded that there was an inverse association between prior RA and AD even after adjusting for potential confounders.

## Introduction

Rheumatoid arthritis (RA) is a prevalent autoimmune disease which primarily affects synovial joints [1]. This disease is characterized by progressive joint damage and bone destruction and can further contribute to joint deformity and severe disability [2,3]. Even though the actual pathophysiology of RA is still unclear, RA is considered to be a multifactorial disease [4]. Many risk factors, including genetic factors, environmental factors, demographic characteristics, etc. have been suspected of being associated with RA [4–6]. Recently, increasing evidence has supported an inflammatory mechanism possibly playing a major role in the development of RA [7,8]. Additionally, inflammation is believed to be etiologically involved in many chronic diseases, such as cardiovascular diseases, metabolic syndrome, mental diseases, neurodegenerative disorders, etc [9–13].

Alzheimer’s disease (AD) is a common neurodegenerative disease which affects approximately 6%~8% of all individuals over 65 years old, and patients with this disease usually experience memory or cognitive impairment [14–16]. Although the genuine mechanisms of AD are still under discussion, many studies have reported that a cytokine-mediated inflammatory pathway is associated with the progression of cognitive impairment and AD [17,18]. Therefore, some of the prior literature investigated the potential association between RA and AD because these two diseases may share similar inflammatory mechanisms. Nevertheless, to date, findings of the previous literature are still controversial, and some methodological limitations were observed in those studies [19–22]. According to studies in the 1990s, patients with RA have a reduced risk of AD; however, recent research showed that RA may increase the risk of cognitive impairment [19–22]. Consequently, in order to clarify this important issue, the aim of this study was to investigate the relationship between prior RA and AD using a large population-based dataset in Taiwan.

## Methods

### Database

Data for this population-based case-control study were sourced from the Taiwan Longitudinal Health Insurance Database 2005 (LHID2005). The LHID2005 includes longitudinal data on original medical records and relevant registration files for 1 million individuals since establishment of the Taiwanese National Health Insurance (NHI) program in 1995. These 1 million individuals were randomly selected from all enrollees listed in the 2005 registry of beneficiaries under the NHI program (*n* = 25.68 million). Numerous investigators have used the LHID2005 to perform observational studies, and a number of studies have been published in international peer-reviewed journals.

### Study Sample

To select AD cases for this study, we initially identified 2283 patients with a diagnosis of AD (ICD-9-CM: 290 and 331.0) from January 2001 to December 2013 who had received prescriptions of acetylcholinesterase inhibitors (AChEIs). In Taiwan, prescribing AChEIs for patients with AD needs to undergo a review procedure which is conducted by a committee in the NHI Administration. This committee consists of neurologists or psychiatrists who evaluate whether those patients are entitled for reimbursement for AChEIs according to patients’ clinical records, cognitive function, biochemistry tests, and diagnostic imaging. We then excluded 12 patients under 50 years of age since this age group has a very low prevalence of AD. Finally, 2271 patients with AD who received AChEIs were included as cases in this study. In addition, we defined the first date of receiving AChEIs as the index date for cases.

The matched controls were extracted from the remaining beneficiaries of the LHID2005. We totally selected 6813 controls (three controls per AD case) from the remaining enrollees matched with cases in terms of sex, age group (50~54, 55~59, 60~64, 65~69, 70~74, 75~79, and ≥80 years), and the year of the index date using the SAS program proc SurveySelect (SAS System for Windows, vers. 9.2, SAS Institute, Cary, NC). For cases, the year of the index date was simply a year when cases first received a prescription for AChEIs. For controls, the year of the index date was a matched year in which the controls utilized medical services. Additionally, we assured that none of the selected controls had a medical history of AD since the beginning of the Taiwanese NHI program in 1995.

### Outcome Measures

In this case-control study, we attempted to demonstrate the association between prior RA and AD. We defined cases with RA based on the ICD-9-CM code 714.0. In Taiwan, a physician may provide a temporary RA diagnosis for subjects who are suspected as RA patients, because physician should conduct associated lab tests or radiography for those subjects for confirmation. If a subject had a confirmed RA diagnosis after the tests, he/ she would receive routine therapy and have a second RA diagnosis in next outpatient visit. Therefore, in order to increase the validity of the RA diagnoses, this study only included the patients who had received two or more RA diagnoses prior to the index date, with at least one being made by a rheumatologist. Additionally, we only recruited RA cases if they had been prescribed at least one type of disease-modifying antirheumatic drug (DMARD). We identified the date of first RA diagnosis as the RA onset date in this case-control study.

### Statistical Analysis

All analyses in this study were conducted with the SAS system (SAS System for Windows, vers. 9.2). Chi-squared tests were used to compare differences in demographic characteristics and comorbidities, including monthly income (≤NT$15,840, 15,841~25,000, ≥25,001), geographic location (northern, central, eastern, and southern Taiwan), urbanization level (five levels, with 1 being the most urbanized and 5 being the least), hypertension, diabetes, hyperlipidemia, stroke, and coronary heart disease between patients with and those without AD. Conditional logistic regressions (stratified on sex, age group, and the year of the index date) were performed to investigate the relationship between AD and prior RA after adjusting for demographic characteristics and comorbidities. The conventional *p*<0.05 was used to estimate statistical significance in this study.

## Results

This population-based study included 2271 patients with AD as cases and 6813 patients without AD as matched controls. The total 9084 patients in this study had a mean age of 76.5±8.2 years. Mean ages for cases and controls were 76.5±7.9 and 76.5±8.3 years, respectively (*p* = 0.960). The demographic characteristics and medical comorbidities of patients with and those without AD are presented in Table 1. There were significant differences in geographic region (*p*<0.001), urbanization level (*p* = 0.002), hypertension (*p*<0.001), diabetes (*p* = 0.027), hyperlipidemia (*p*<0.001), stroke (*p*<0.001), and coronary heart disease (*p*<0.001) between cases and controls after matching for sex, age group, and the year of the index date. There was no significant difference in monthly income between cases and controls.

**Table 1:** Demographic characteristics and medical comorbidities of patients with Alzheimer’s disease (AD) and controls in Taiwan (*n* = 9084).

Variable	Patients with AD *n =* 2271		Controls *n =* 6813		*p* value
	Total no.	Column %	Total no.	Column %	
Age (years)					1.000
50~59	72	3.2	216	3.2	
60~64	120	5.3	360	5.3	
65~69	210	9.3	630	9.3	
70~74	435	19.2	1,305	19.2	
75~79	549	24.2	1,647	24.2	
≥80	885	39.0	2,655	39.0	
Male	883	38.9	2,649	38.9	1.000
Hypertension	1,514	66.7	4,971	73.0	<0.001
Diabetes	694	30.6	2,253	33.1	0.027
Hyperlipidemia	806	35.5	3,000	44.0	<0.001
Stroke	722	31.8	1,788	26.2	<0.001
Coronary heart disease	676	29.8	2,319	34.0	<0.001
Monthly income					0.094
≤NT$15,840	1,374	60.5	4,143	60.8	
NT$15,841~25,000	789	34.7	2,271	33.3	
≥NT$25,001	108	4.8	399	5.9	
Geographical region					
Northern	838	36.9	2,859	42.0	<0.001
Central	635	28.0	1,680	24.7	
Southern	744	32.8	2,034	29.9	
Eastern	54	2.4	240	3.5	
Urbanization level					0.002
1 (most urbanized)	630	27.7	1,692	24.8	
2	587	25.9	1,890	27.7	
3	374	16.5	999	14.7	
4	333	14.7	1,032	15.2	
5 (least urbanized)	347	15.3	1,200	17.6	

The average exchange rate in 2014 was US$1.00≈New Taiwan (NT)$30.

Table 2 shows the prevalence of prior RA between cases and controls. The findings reveal that 330 (3.63%) of all selected patients had an RA diagnosis before the index date. Prior RA was found in 60 (2.64%) cases and in 270 (3.96%) controls. In addition, a Chi-squared test found that the patients with AD had a significantly lower prevalence of prior RA compared to those without AD (*p* = 0.004).

**Table 2:** Association between Alzheimer’s disease (AD) and prior rheumatoid arthritis (RA) (*n* = 9084).

Presence of prior RA	Total study sample *n =* 9084		Patients with AD *n =* 2271		Controls *n =* 6813		*p* value
	No.	%	No.	%	No.	%	
Yes	330	3.63	60	2.64	270	3.96	0.004
No	8754	96.37	2211	97.36	6543	96.04	

Table 3 displays the crude odds ratios (ORs), adjusted ORs, and 95% confidence intervals (CIs). The conditional logistic regression analysis (stratified by sex, age group, and year of the index date) indicated that the crude OR of prior RA for cases was 0.66 (95% CI: 0.49~0.87) compared to controls. After adjusting for patients’ geographic location, urbanization level, and comorbidities, the adjusted OR of prior RA for cases was 0.73 (95% CI: 0.55~0.98) compared to those patients without AD. Additionally, it is noteworthy that RA was negatively associated with the medical comorbidities of hypertension (adjusted OR = 0.77, 95% CI = 0.69~0.86), hyperlipidemia (adjusted OR = 0.73, 95% CI = 0.65~0.81), and coronary heart disease (adjusted OR = 0.86, 95% CI = 0.77~0.96). However, RA was significantly associated with the occurrence of stroke (adjusted OR = 1.45, 95% CI = 1.30~1.62).

**Table 3:** Crude and adjusted odds ratios (ORs) of prior rheumatoid arthritis (RA) among sampled patients.

Variables	Patients with Alzheimer’s disease vs. Controls	
	Crude OR (95% CI)	Adjusted OR (95% CI)
Prior RA	0.66** (0.49~0.87)	0.73* (0.55~0.98)
Monthly income		
≤NT$15,840	1.00	1.00
NT$15,841~25,000	1.04 (0.94~1.16)	1.11 (0.98~1.25)
≥NT$25,001	0.79 (0.62~1.00)	0.80 (0.63~1.02)
Urbanization level		
1	1.00	1.00
2	0.83** (0.73~0.95)	0.74*** (0.65~0.85)
3	1.01 (0.87~1.17)	0.85* (0.72~0.99)
4	0.87 (0.74~1.01)	0.68*** (0.57~0.81)
5	0.78*** (0.67~0.90)	0.60*** (0.50~0.72)
Geographic region		
Northern	1.00	1.00
Central	1.29*** (1.15~1.45)	1.44*** (1.26~1.65)
Southern	1.25*** (1.12~1.40)	1.33*** (1.18~1.51)
Eastern	0.77 (0.57~1.04)	0.93 (0.67~1.27)
Hypertension	0.73*** (0.66~0.81)	0.77*** (0.69~0.86)
Diabetes	0.89* (0.80~0.99)	1.01 (0.91~1.13)
Hyperlipidemia	0.70*** (0.63~0.77)	0.73** (0.65~0.81)
Stroke	1.32*** (1.19~1.47)	1.45*** (1.30~1.62)
Coronary heart disease	0.82*** (0.74~0.91)	0.86** (0.77~0.96)

Note:

* *p*<0.05

** *p*<0.01

*** *p*<0.001.

The adjusted odds ratios were derived from a conditional logistic regression model and adjusted for all other variables.

The average exchange rate in 2014 was US$1.00≈New Taiwan (NT)$30.

## Discussion

This population-based case-control study found an inverse association between prior RA and AD. The OR of prior RA for patients with AD was 0.73 (95% CI: 0.55~0.98) compared to those patients without AD after adjusting for patients’ geographic location, urbanization level, and comorbidities. To the best of our knowledge, only a few studies [7,8,17,18] have attempted to investigate the potential association between prior RA and AD, even though these two diseases supposedly share similar pathological pathways.

The negative relationship between prior RA and AD revealed by this study is consistent with previous studies in the 1990s [19–21]. One study in the United Kingdom, which included 96 patients with AD and 92 controls, observed a significant inverse association between AD and RA (*p*<0.005) [19]. Other research also found that the prevalence of AD in patients with RA was unexpectedly lower than that in the general population [20]. Additionally, a study in Finland displayed that only two individuals (1.2‰) died of AD among patients with RA, but 227 individuals (5.4‰) died of AD in the entire population [21]. Conversely, results of other studies conflict with our results. For instance, an increased risk of cognitive impairment in patients with RA was reported by Wallin et al. [22]. In addition, a meta-analysis which involved three cohort studies and two cross-sectional studies reported a significantly elevated risk of all subtypes of dementia among patients with RA, and the pooled risk ratio in that study was 1.61 (95% CI, 1.10~2.37) [22–25]. These controversial findings may have been due to several methodologic limitations and the definitions of study cases. For example, using a self-administered questionnaire to identify RA cases might be less accurate than using diagnostic codes provided by physicians [22]. Additionally, most of the prior literature included all subtypes of dementia as study cases [22–25]. However, our study only selected patients with AD, which is recognized as one of the common forms of dementia, as study cases.

The actual mechanisms of the relationship between prior RA and AD still remain unclear. The lower odds of prior RA in patients with AD than those without AD is thought to be associated with the use of non-steroidal anti-inflammatory drugs (NSAIDs) or the upregulation of granulocyte macrophage colony-stimulating factors (GM-CSFs) in patients with RA. According to the prior literature, the use of NSAIDs in patients with RA was assumed to possibly prevent the incidence and development of AD [20,26–28]. A recent meta-analysis also reported that the use of NSAIDs was significantly associated with a decreased risk of AD (relative risk = 0.72, 95% CI = 0.62~0.84) [29]. Nevertheless, one large randomized controlled trial found that the use of NSAIDs could not prevent the incidence of AD among dementia-free subjects [30]. It is still uncertain whether the use of NSAIDs plays a role in the underlying mechanism. Consequently, intrinsic factors within the RA pathogenesis are suspected of contributing to protective effects against the occurrence of AD [31]. To date, increasing biological evidence further supports GM-CSFs, which are highly produced in synovitis during RA pathogenesis, possibly reversing amyloidosis and cognitive impairment in AD [31–34].

The principle strength of this case-control study is the use of a large population-based database with high health benefit coverage in Taiwan. The sample size of this study was much larger than previous studies which investigated the association between RA and AD. The LHID2005 could provide an adequate sample size and increase the statistical power for the results. The feature of this database may also reduce the selection bias of the findings, which commonly occurs in observational studies. Additionally, the LHID2005 used in this study includes all medical records regarding diagnoses, therapeutic procedures, and medications since patients were enrolled in the NHI program in Taiwan. This characteristic of the LHID2005 can avoid a recall bias which frequently occurs in case-control studies.

Nevertheless, several limitations should be taken into consideration. First, some lifestyle information, laboratory records on inflammatory biomarkers, family history, and genetic factors are not available in the LHID2005. These factors are considered to affect cognitive function and might confound the association between prior RA and AD [35–37]. Second, the LHID2005 provides no detailed records of biochemical tests, Mini-Mental State Examination (MMSE) scores, or medical imaging. Thus, some researchers may criticize the accuracy of the AD diagnosis. However, we identified patients receiving AChEIs as AD cases in this study. In Taiwan, prescribing AChEIs for patients with AD undergoes a review procedure conducted by a committee. This committee evaluates whether those patients are allowed reimbursement for AChEIs according to the patients’ clinical symptoms, biochemical tests, MMSE scores, and medical imaging with computed tomography (CT) scans or magnetic resonance imaging (MRI) scans. Finally, the overwhelmingly majority of patients recruited in this study were of Chinese ethnicity, so the ability to generalize the findings to other ethnic groups is uncertain.

In summary, this population-based case-control study found that prior RA was negatively associated with AD even after adjusting for patients’ demographics and comorbidities. Further large epidemiologic studies are still required to clarify the relationship between prior RA and AD in different ethnic groups and countries. Future experimental studies are warranted to determine the actual mechanisms for the inverse association between prior RA and AD.
